# 

**Published:** 1889-04-13

**Authors:** 


SATURDAY, APRIL 13th, 1889.

"The Enthusiasm of Humanity" - - - - - - 17
**Strychnia in Snakebite** - - - - - - - - 18
**The Doctor's Pulpit.**—Man and his Enemies - - - 19
**The Student's Column.**—"A Textbook of Pathological Anatomy and Pathogenesis" - - - - - - - - 20
**Variations in Hospital Expenditure.**—I. - - - 21
**Within the Wards.**—Vivisectors and their Work - - 22
**Glances at the Insane in other Lands.**—VIII. - - 23
**Notes and News** - - - - - - - - - 24
**Everybody's Page** - - - - - - - - - 26
**Annotations.**—New "Tight-lace Diseases"—Skim Milk as Food—Hath Music Charms - - - - - - - 27
**Practical Socialism** - - - - - - - - 28
**"Her Heart's Mistake."** By E. D. Cumming, Author of "An Ordeal by Silence" - - - - - - - - 29
**Scraps and Gleanings** - - - - - - - 32
**Amusements and Relaxation** - - - - - - 32

### EXTRA SUPPLEMENT—THE NURSING MIRROR.

**En Passant.**—Lady Nurses—Women and Dress—Missionaries and Massage in India—Pauper Nurses—National Pension Fund for Nurses—"A Fellow Feeling" - - - - v
**Nursing in the Soudan**—Going into Action - - - - vi
**The Nursing and Management of the Insane.** By T. Duncan Greenlees, M.B. - - - - - - - vi
**Work to Do** - - - - - - - - - - vii
**Everybody's Opinion.**—The Pension Fund—Nursing the Insane - - - - - - - - - - - viii
**Vacancies** - - - - - - - - - - - viii
**Notes and Queries** - - - - - - - - - viii
**Songs of Golden Deeds** - - - - - - - - viii

